# Occupational Therapy in Acute Branch Atheromatous Disease With Frequent Motor Paralysis Fluctuations: A Case Report

**DOI:** 10.7759/cureus.91912

**Published:** 2025-09-09

**Authors:** Takaya Matsubara, Takashi Hoei, Kodai Miyara, Shuji Matsumoto

**Affiliations:** 1 Department of Rehabilitation, Nadogaya Hospital, Kashiwa, JPN; 2 Department of Rehabilitation, Kagoshima University Hospital, Kagoshima, JPN; 3 Department of Rehabilitation, Kyushu University of Nursing and Social Welfare, Tamana, JPN; 4 Center for Medical Sciences, Ibaraki Prefectural University of Health Sciences, Ami, JPN

**Keywords:** acute stroke, branch atheromatous disease, neuromuscular electrical stimulation, occupational therapy, paralysis, rehabilitation, repetitive facilitation exercise, upper limb function

## Abstract

Branch atheromatous disease (BAD) often progresses during the acute phase. During acute rehabilitation, clinicians should prioritize monitoring neurological symptom progression. We treated a patient with right-sided, finger-limited motor paralysis and frequent transient motor paralysis fluctuations due to BAD by implementing early upper-limb occupational therapy while maintaining rigorous risk management. Occupational therapy included standard upper limb functional training, continuous neuromuscular electrical stimulation, and repetitive facilitation exercises. During occupational therapy, blood pressure was carefully monitored, and neurological symptoms were regularly assessed. Any symptom changes were promptly communicated among occupational therapists, rehabilitation specialists, and neurosurgeons. Although transient motor paralysis occurred five times, timely treatment, including medication adjustments, prevented further neurological deterioration. Additionally, upper-limb function and object manipulation ability improved over time. Risk management is critically important in occupational therapy for acute BAD. This case demonstrated that repetitive facilitation exercises combined with continuous neuromuscular electrical stimulation can be safely initiated in the early stages of stroke, potentially improving upper limb dysfunction.

## Introduction

Branch atheromatous disease (BAD) is a pathological condition characterized by atherosclerotic-induced stenosis or occlusion near large-diameter perforating branches of parent arteries, leading to infarction throughout the perforating branch [1,2]. Clinically, BAD presents significant challenges, including progression during the acute phase, poor response to medical treatment [3], and limited recovery of motor paralysis during convalescent rehabilitation [4]. In the acute phase of cerebral infarction, impaired cerebral blood flow autoregulation may render the brain vulnerable to ischemic damage from even minor blood pressure fluctuations or hemodynamic changes [5]. Given these findings, rigorous management and early implementation of upper limb functional exercises are critical in occupational therapy for acute BAD-related upper limb dysfunction. However, due to symptom fluctuations, few reports have addressed occupational therapy for acute BAD, and evidence-based interventions remain undefined.

Repetitive facilitative exercise (RFE), endorsed by the 2021 Japanese Stroke Treatment Guidelines (revised 2023) [6,7], has demonstrated efficacy in improving upper limb function. A randomized controlled trial by Shimodozono et al. [8] reported significant outcomes with RFE compared to conventional exercises in patients with mild-to-moderate upper limb paralysis. RFE involves selecting a target movement pattern in supine or seated position, combining patient-generated voluntary effort with therapist-assisted manual facilitation, and high-frequency repetition of these movements, allowing minimal blood pressure fluctuations (critical in acute BAD), feasibility in supine positioning (expanding movement options), and enhanced muscle contraction efficacy. These features have established RFE as an effective facilitation technique for improving hemiplegia. Therefore, its supine application minimizes blood pressure fluctuations from postural changes in patients with acute BAD, thereby demonstrating strong clinical applicability for upper limb rehabilitation. Recent studies further indicated that combining RFE with continuous low-amplitude neuromuscular electrical stimulation (cNMES) significantly enhances treatment efficacy [9-11].

We present a case of recurrent right finger motor paralysis exacerbations due to BAD. We hypothesized that early occupational therapy featuring cNMES-combined RFE would prevent progressive deterioration of neurological function by minimizing blood pressure fluctuations. Despite the patient’s fluctuating finger motor paralysis secondary to cerebral infarction, we successfully implemented early occupational therapy while maintaining stringent risk management protocols.

## Case presentation

**Figure 1:**
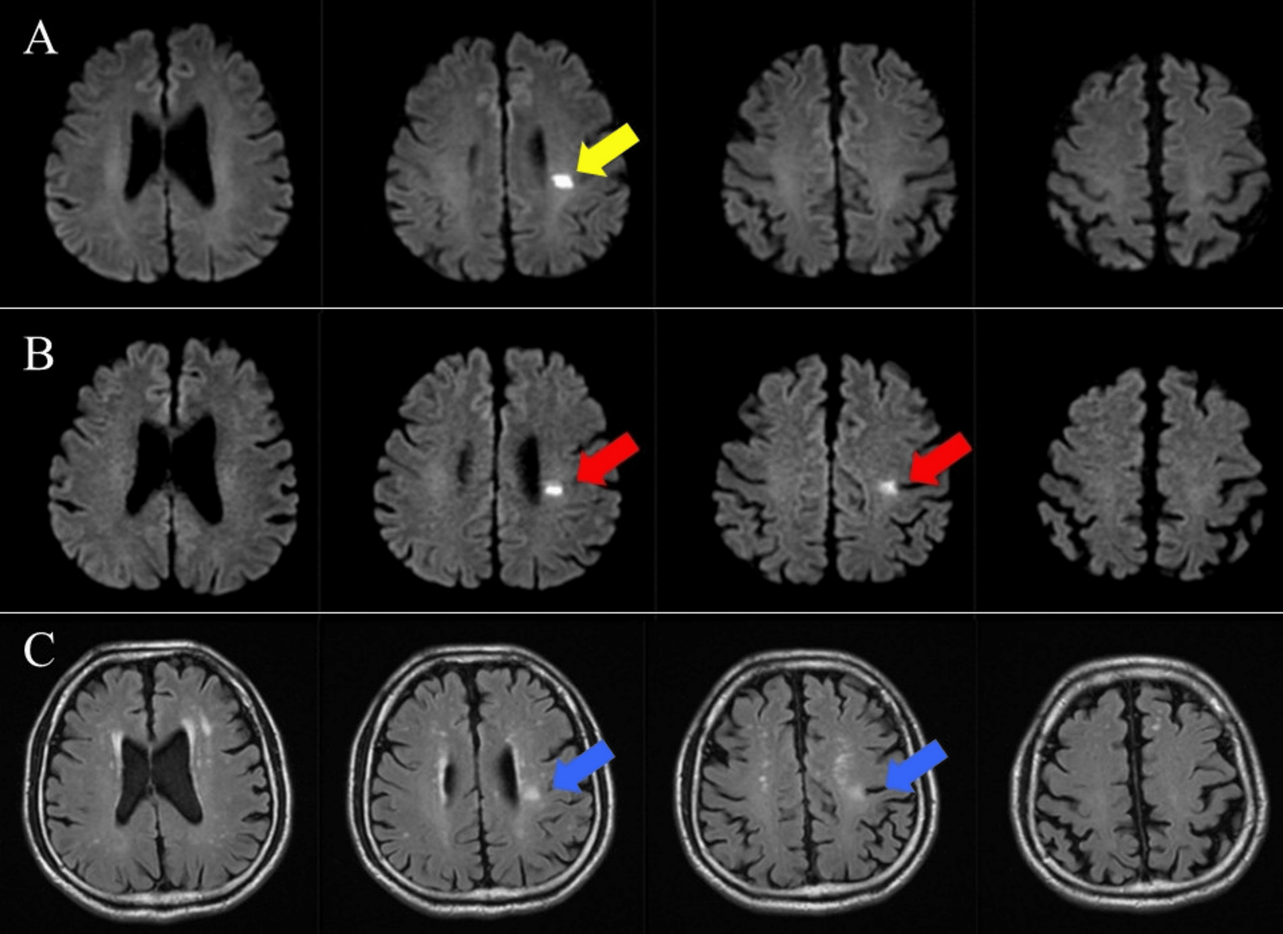
MRI of the patient. (A) MRI-DWI. Day 1: Infarction of the left corona radiata as indicated by the yellow arrow. (B) MRI-DWI. Day 2: Infarction in the left corona radiata progressed to two slices. Enlargement of the infarct area was noted, as indicated by red arrows. (C) MRI-FLAIR. Day 11: Left corona radiata infarction is identified in two slices (blue arrows). MRI: magnetic resonance imaging; DWI: diffusion-weighted imaging; FLAIR: fluid-attenuated inversion recovery.

A 56-year-old right-handed man presented to our hospital with right finger weakness. He had no history of stroke risk factors in his personal or family medical history. Brain magnetic resonance imaging (MRI) and diffusion-weighted imaging (DWI) revealed a hyperintense lesion in the left corona radiata (Figure 1A), consistent with lacunar infarction. The patient was hospitalized and started on antiplatelet therapy (ozagrel sodium, 80 mg/day). On hospital day two, his right finger weakness progressed, with follow-up MRI-DWI demonstrating infarct expansion (Figure 1B), prompting reclassification as BAD. Treatment was intensified to dual therapy, including anticoagulant therapy (argatroban, 10 mg/day, increased to 20 mg/day from illness day four) and antiplatelet therapy (clopidogrel, 75 mg/day). Blood volume expansion therapy (low-molecular-weight dextran for seven days) was added on illness day seven. Occupational therapy was initiated at the bedside on post-stroke day two and was changed to occupational therapy in the rehabilitation room on post-stroke day four. At the initiation of occupational therapy, the patient's functional status was either independent or modified independently in activities of daily living (ADL), with a functional independence measure (FIM) score of 104 (unassessed items: bathtub transfers and stair climbing). No sensory deficits were observed, and upper limb function was assessed using the Stroke Impairment Assessment Set-Motor (SIAS; maximum score: 5 points), yielding a score of 5 points on the knee test, 4 on the finger test, and a negative Barré sign, consistent with motor paralysis limited to the fingers.

Occupational therapy was provided once daily, five times per week, from post-stroke day two until day 21 of transfer to the rehabilitation hospital, with each 40-minute session focusing on RFE combined with cNMES. Before initiating occupational therapy, we verified medication usage by reviewing medical records and cross-referencing physician and nursing documentation to confirm consistency. Neurological symptoms were closely monitored throughout the intervention. During therapy sessions, patients were asked to report subjective symptoms related to paralysis, including ease of finger flexion and perceived weakness. Blood pressure was monitored at the start of each session and during postural changes. Changes in motor paralysis were evaluated using the Barré and SIAS finger tests.

RFE was performed by selecting multiple movement patterns (flexion and extension of the forearm, wrist, fingers, and individual fingers), as described by Kawahira et al. [12], with each pattern repeated 100 times. Extension patterns (forearm, wrist, hand, and individual fingers) were performed in the supine position, while flexion patterns (individual fingers) were performed in the sitting position. cNMES was performed using a low-frequency therapy device (ESPURGE®, Ito Ultrasonic Co., Ltd., Tokyo, Japan). Electrodes were placed bilaterally (bipolar method) over the motor points of the target muscles for each RFE movement pattern. Stimulation parameters were set at 50 Hz frequency, 250 μs pulse width, and motor threshold intensity (minimal intensity required to elicit muscle contraction without joint movement) [9,13]. Outcome measures included upper limb function using the Fugl-Meyer assessment (FMA) upper limb motor items and SIAS knee-oral and finger tests, manual dexterity using the simple test for evaluating hand function (STEF), and ADL using the FIM. Assessments were conducted at baseline (hospital day five) and upon transfer to another hospital (hospital day 21). The SIAS knee-mouth test, finger function test, and FIM were additionally performed on occupational therapy day two as part of the initial evaluation. The patient provided written informed consent for both the intervention and publication of this report.

**Figure 2:**
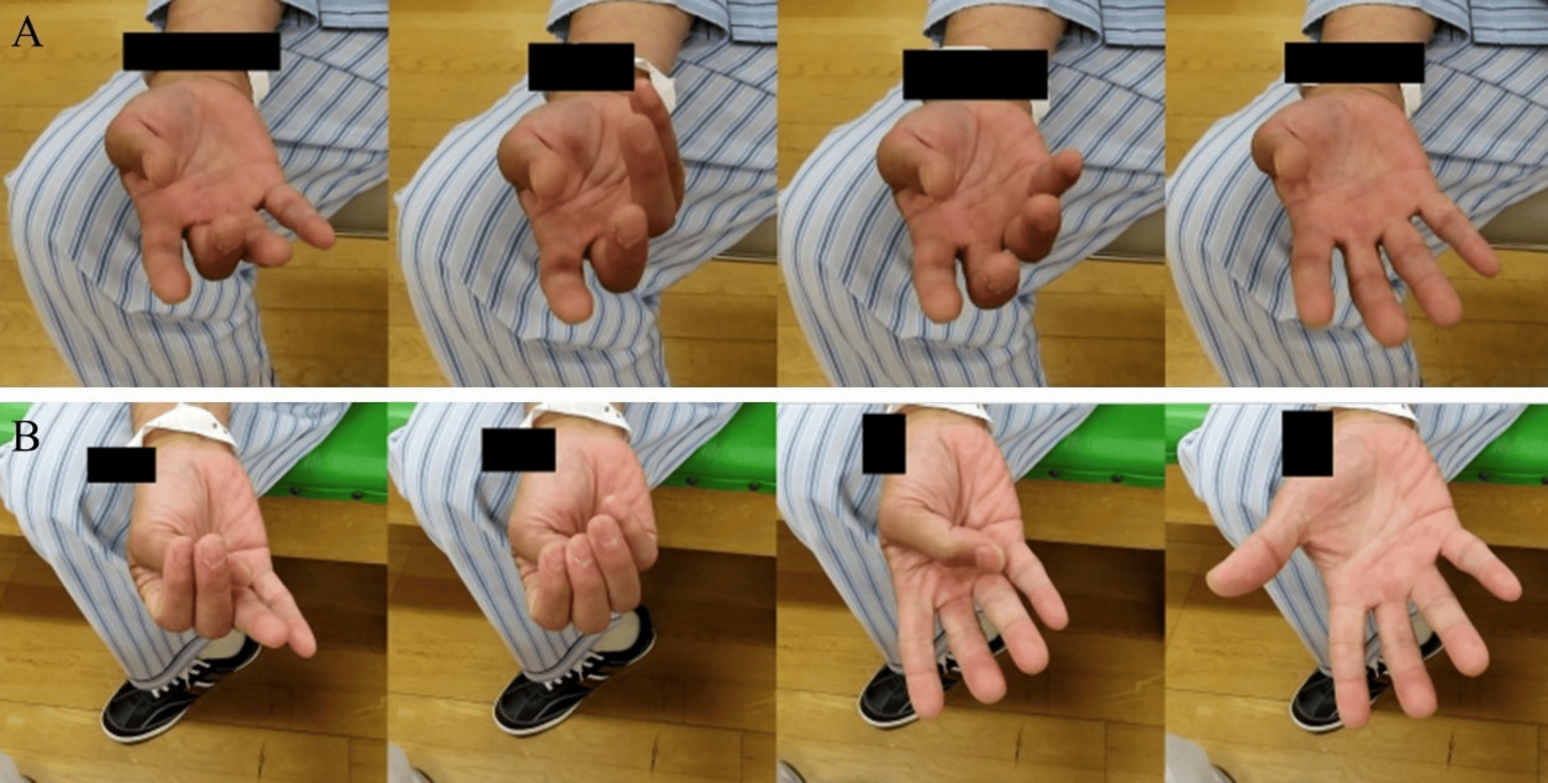
SIAS finger test (comparison of the same movement) before and after transient fluctuations that occurred on the 15th day of illness. In the Stroke Impairment Assessment Set (SIAS) test, the fingers are flexed sequentially from thumb to little finger and then extended sequentially from little finger to thumb. (A) During transient symptom fluctuation. (B) After 10 minutes, when symptoms recovered.

Motor paralysis fluctuations occurred on five occasions (days 2, 7, 10, 12, and 15). On occupational therapy day 15 in the rehabilitation room, a significant transient progression of motor paralysis was observed, manifesting a decrease in the SIAS finger test score from 4 to 2. All symptoms resolved within 10 minutes following appropriate interventions (Figure 2). As detailed in Table 1, which summarizes functional assessment changes, no progressive neurological deterioration occurred, and upper limb function showed gradual improvement. Specifically, total FMA score improved from 56 (initial) to 61 (final), reflecting a five-point improvement in wrist and finger function; SIAS finger test score increased from 4 to 5; and STEF score increased from 78 to 93. For ADL, the FIM-motor score improved from 70 to 89, and the FIM-cognitive score improved from 34 to 35. The FIM-total scores improved from 104 to 124, with self-care items, including eating, grooming, dressing, and bathing, progressing from modified to complete independence.

**Table 1:** Evaluation of the upper limb function, object manipulation ability, and ADL. ADL: activities of daily living; FMA: Fugl-Meyer assessment; SIAS: Stroke Impairment Assessment Set; STEF: simple test for evaluating hand function; FIM: Functional Independence Measure.

Evaluation items		Initial	Final evaluation (day 21)
FMA upper limb motor items (total score)	Points	56	61
Shoulder/elbow/forearm, coordination/speed	Points	42	42
Wrist/hand	Points	14	19
SIAS knee-mouth test		5	5
SIAS finger function test		4	5
STEF	Points	78	93
FIM-motor score	Points	70	89
FIM-cognitive score	Points	34	35
FIM-total score	Points	104	124

All five episodes of motor paralysis (occurring between illness day two to day 15) resolved within 10 minutes. Follow-up MRI on illness day 11 showed no infarct expansion compared to day two (Figure 1C). For symptom management, during ward episodes, the patient was instructed to lie supine and notify the nursing staff. During rehabilitation sessions, the patient was maintained in a supine position and promptly evaluated by a rehabilitation physician, with findings communicated to the neurosurgeon.

On illness day 15, a pronounced motor paralysis fluctuation occurred (Figure 2A). The systolic and diastolic blood pressures were recorded at 156 and 97 mmHg, respectively, before initiating occupational therapy. During motor paralysis fluctuation, the systolic blood pressure decreased to 139 mmHg, and the diastolic blood pressure decreased to 90 mmHg, reflecting an overall decline in blood pressure. The patient was immediately placed in a supine position and instructed to rest; a rehabilitation physician was called for evaluation. After resting in the supine position, systolic blood pressure returned to 161 mmHg, and diastolic blood pressure returned to 94 mmHg, with symptomatic improvement observed within 10 minutes (Figure 2B).

## Discussion

In acute cases presenting with motor paralysis limited to the fingers due to BAD and frequent fluctuations in transient motor paralysis, we implemented early occupational therapy centered on RFE under cNMES, with careful attention to risk management. Although transient fluctuations persisted during treatment, no progressive worsening occurred. Improvements in upper limb function, object manipulation ability, and ADL were achieved through early occupational therapy intervention, leading to a favorable outcome.

Hoshino [3] reported that approximately half of all cases of BAD with infarction of the lenticulostriate artery and paracentral bridge artery are susceptible to progressive cerebral infarction and demonstrate poor acute-phase outcomes. In this case, infarction of the lenticulostriate artery was observed, indicating a high risk for progressive cerebral infarction from disease onset. In this case, by thoroughly assessing patients’ condition before occupational therapy intervention, monitoring blood pressure during sessions, and evaluating neurological symptoms, we promptly detected transient fluctuations while accounting for the potential progressive worsening of BAD. Early neurological deterioration in BAD often occurs within two to three days; this case was a rare instance where fluctuations recurred five times over a prolonged period. No established treatment for BAD existed; however, drug monotherapy has limitations in preventing disease progression and improving functional outcomes. Therefore, aggressive treatment with combination therapy is being considered [14]. In this case, multiple drug therapies were administered. Following transient symptom fluctuations, rehabilitation physicians and neurosurgeons collaborated, facilitating timely and appropriate treatment adjustments. Additionally, occupational therapy involves prolonged interaction between patients and therapists, allowing close observation of changes in upper limb function and subjective symptoms. By sharing these observations with a multidisciplinary team, we propose that this process contributes to informing physicians’ treatment decisions.

The transient fluctuation in severe motor paralysis observed on day 15 in this case was potentially associated with impaired cerebral autoregulation. During the acute phase of cerebral infarction, cerebral autoregulation is impaired, rendering the brain vulnerable to hemodynamic changes that may exacerbate neurological symptoms [15]. Additionally, in cerebral infarction, a core region of complete ischemia surrounded by a potentially salvageable penumbral region is noted [16]. According to the 2019 Japanese guidelines for the treatment of hypertension, patients with acute- or hyperacute-phase cerebral infarction (within two weeks of onset) who are not eligible for thrombolytic therapy should receive careful antihypertensive therapy only if systolic blood pressure remains ≥ 220 mmHg or diastolic blood pressure remains ≥ 120 mmHg, or if cardiovascular or renal complications are present; additionally, these guidelines suggests that hypotension may expand the infarct core and penumbra [17]. The transient symptom fluctuation observed on day 15 in this case may have occurred because it coincided with the recent cessation of hemodilution therapy, whereby even a slight blood pressure reduction associated with sitting could have significantly affected cerebral perfusion. These findings suggest that during occupational therapy for acute cerebral infarction, clinicians must carefully monitor blood pressure, particularly for decreases, while remaining vigilant for potential neurological fluctuations. Notably, early neurological worsening is recognized as a poor prognostic factor in BAD [18,19], necessitating close monitoring of neurological symptom fluctuations. However, Tokuda et al. [19] reported that while blood pressure and symptoms should be monitored in the sitting position in patients with BAD, early mobilization and active occupational therapy may be initiated, provided risk management is implemented.

Early occupational therapy intervention in this case resulted in improvements of five points on the total FMA score, 15 points on the STEF, and 20 points on the FIM-total score, demonstrating a certain degree of improvement. The minimal clinically important difference (MCID) for the FMA upper limb motor items is 4 points, suggesting this case achieved sufficient change. Regarding STEF, while there is no established MCID or minimal detectable change (MDC), it showed strong correlations with FMA, Action Research Arm Test (ARAT), and Motor Activity Log (MAL). Since STEF specifically focuses on assessing finger dexterity compared to ARAT, the fact that STEF improvement was achieved suggests a favorable course. The STEF score of 93 at the final evaluation for this case was within the normal range for STEF in the same age group (maximum 100 points, minimum 92 points: average 98 points). Regarding FIM, the MCID for the FIM motor function score was 17. Since the improvement value for this case is 19, it can be considered a clinically meaningful improvement.

In this study, we implemented occupational therapy involving RFE and cNMES for acute-phase BAD patients based on the concept of minimizing blood pressure fluctuations. Previous reports suggest that RFE combined with cNMES can improve upper limb function in acute-phase cerebral infarction [8]. The most critical aspect of performing active occupational therapy was monitoring symptom fluctuations while collaborating across disciplines to manage risks. In our case, although transient fluctuations in motor paralysis occurred, no progressive neurological deterioration was observed, enabling early upper limb function training. Because RFE under cNMES involves multiple movement patterns performed primarily in the supine position (with some in sitting), it facilitates blood pressure management in patients with BAD while allowing high-frequency execution of target movements. We propose that RFE under cNMES, which actively elicits muscle contraction, may be an effective intervention for early upper limb function training in acute-phase occupational therapy. However, this study is limited to its single-case design and descriptive nature. Future research should expand to larger case series to evaluate the safety and efficacy of early upper limb functional exercises in BAD.

## Conclusions

In our case, where BAD induced localized motor paralysis in the right hand, accompanied by frequent transient motor paralysis fluctuations, we performed early occupational therapy while closely monitoring risk management. Although five episodes of symptom fluctuation occurred, no progressive neurological deterioration was noted, and the patient achieved improvements in upper limb function, object manipulation ability, and ADL, leading to a favorable outcome. RFE under cNMES could be useful for upper limb functional training during the acute phase of BAD. However, this study was limited to a single case report, necessitating further investigation for broader validation.
